# Efficacy of motivating short interventions for smokers in primary care (COSMOS trial): study protocol for a cluster-RCT

**DOI:** 10.1186/s13063-018-3071-z

**Published:** 2019-01-25

**Authors:** Thomas Grischott, Oliver Senn, Thomas Rosemann, Anja Frei, Jacques Cornuz, Eva Martin-Diener, Stefan Neuner-Jehle

**Affiliations:** 1 Institute of Primary Care, University and University Hospital of Zurich, Pestalozzistrasse 24, CH-8091 Zurich, Switzerland; 2 Epidemiology, Biostatistics and Prevention Institute, University of Zurich, Hirschengraben 84, CH-8091 Zurich, Switzerland; 3 Department of Ambulatory Care and Community Medicine, Rue du Bugnon 44, CH-1011 Lausanne, Switzerland

**Keywords:** Smoking cessation, Health behaviour, Health promotion, Counselling, Motivational interviewing, Shared decision-making, Patient-centredness, Primary care

## Abstract

**Background:**

Tobacco abuse is a frequent issue in general practitioners' (GPs') offices, with doctors playing a key role in promoting smoking cessation to their patients. However, not all smokers are ready and willing to give up smoking. Thus, a GP focusing on smoking cessation alone might waste the opportunity to improve his patient’s health by supporting a change in another harmful behaviour pattern. The aim of this study is to determine whether multi-thematic coaching will lead to higher overall health benefits without resulting in a reduced rate of successful smoking cessations, compared with a monothematic smoking cessation approach.

**Methods:**

The study is designed as a two-armed, double-blinded, cluster-randomised trial. GPs will be randomly assigned to the intervention or control group. In the intervention group, GPs will undergo training in patient-centred coaching, shared decision-making and motivational interviewing. The control group will be trained in a state-of-the-art smoking cessation algorithm.

GPs will approach adult cigarette-smoking patients and advise those included according to the GP’s group affiliation. The primary outcome is the between-group difference in the proportion of participants who achieve a beneficial change in at least one of seven different health-related behavioural dimensions, 12 months post baseline. Secondary outcomes include smoking cessation rates and the patients’ self-perceived smoking-related motivation, self-efficacy and planning behaviour. Additionally, covariates describing both GPs and patients will be collected before the start of the intervention, and process outcome measures in compliance with the RE-AIM (Reach Effectiveness Adoption Implementation Maintenance) framework will be recorded during the ongoing study.

**Discussion:**

Tobacco consumption is still highly prevalent in the general population and often goes hand in hand with other behaviour patterns with adverse health effects. This study will add to the literature regarding effective strategies available to GPs to address unhealthy behaviour among their smoking patients beyond mere smoking cessation counselling. The study will also establish a basis for decisions about further promotion and dissemination of the coaching under study.

**Trial registration:**

ISRCTN, ISRCTN38129107. Registered on 2 October 2017.

**Electronic supplementary material:**

The online version of this article (10.1186/s13063-018-3071-z) contains supplementary material, which is available to authorized users.

## Background

Tobacco use is a frequent issue in general practitioners' (GPs') offices, with doctors playing a key role in promoting smoking cessation to their smoking patients [1]. For this purpose, standardised brief interventions involving both counselling and supporting drug therapy have been established and evaluated for the primary care setting over the last years [2, 3].

However, not all smokers are ready and willing to give up smoking. Therefore, up-to-date stop-smoking programmes are based on the transtheoretical model of behaviour change [4] and thus take the patients’ readiness to stop smoking [3, 5] into account.

The patient-centred “Health Coaching” programme [6], developed by the Swiss College for Primary Care Medicine [7], is based on similar principles. Remarkably, this programme does not focus on smoking cessation or any other single health behaviour alone but includes several health-related behavioural dimensions in parallel. Particular emphasis is put on the patient-driven choice of which topic to address. The Health Coaching programme has proved its efficiency and practicability in a clinical trial carried out within the setting of primary care practitioners in eastern Switzerland [8].

Combining state-of-the-art smoking cessation counselling with the Health Coaching approach, exploring patients’ motivation for smoking cessation can be expanded into a more comprehensive exploration of their readiness to improve their health in any prioritised topic, hopefully leading to additional benefits beyond those gained from tobacco abstinence. We assume that training the GPs’ communication skills and incorporating elements of shared decision-making and motivational interviewing into the counselling process will lead to higher overall health benefits for smokers. Importantly, this broader perspective of health behaviour changes should not be paid for in lower smoking cessation rates in comparison with patients exposed to an intervention tailored to smoking cessation alone.

### Study hypothesis

We hypothesise that multidimensional and patients’ motivation-driven brief interventions by GPs improve several relevant health outcomes of smokers. We assume that these improvements surpass the beneficial effects of monothematic state-of-the-art smoking cessation counselling, and we further assume that the smoking cessation rates achievable through the novel interventions are not inferior to those known from state-of-the-art smoking cessation counselling.

To verify these hypotheses, we plan to train the GPs in the intervention group in how to coach smokers according to the Health Coaching programme [6], while the control group GPs will undergo training in monothematic smoking cessation counselling [5].

## Methods/design

### Study design and setting

**Fig. 1:**
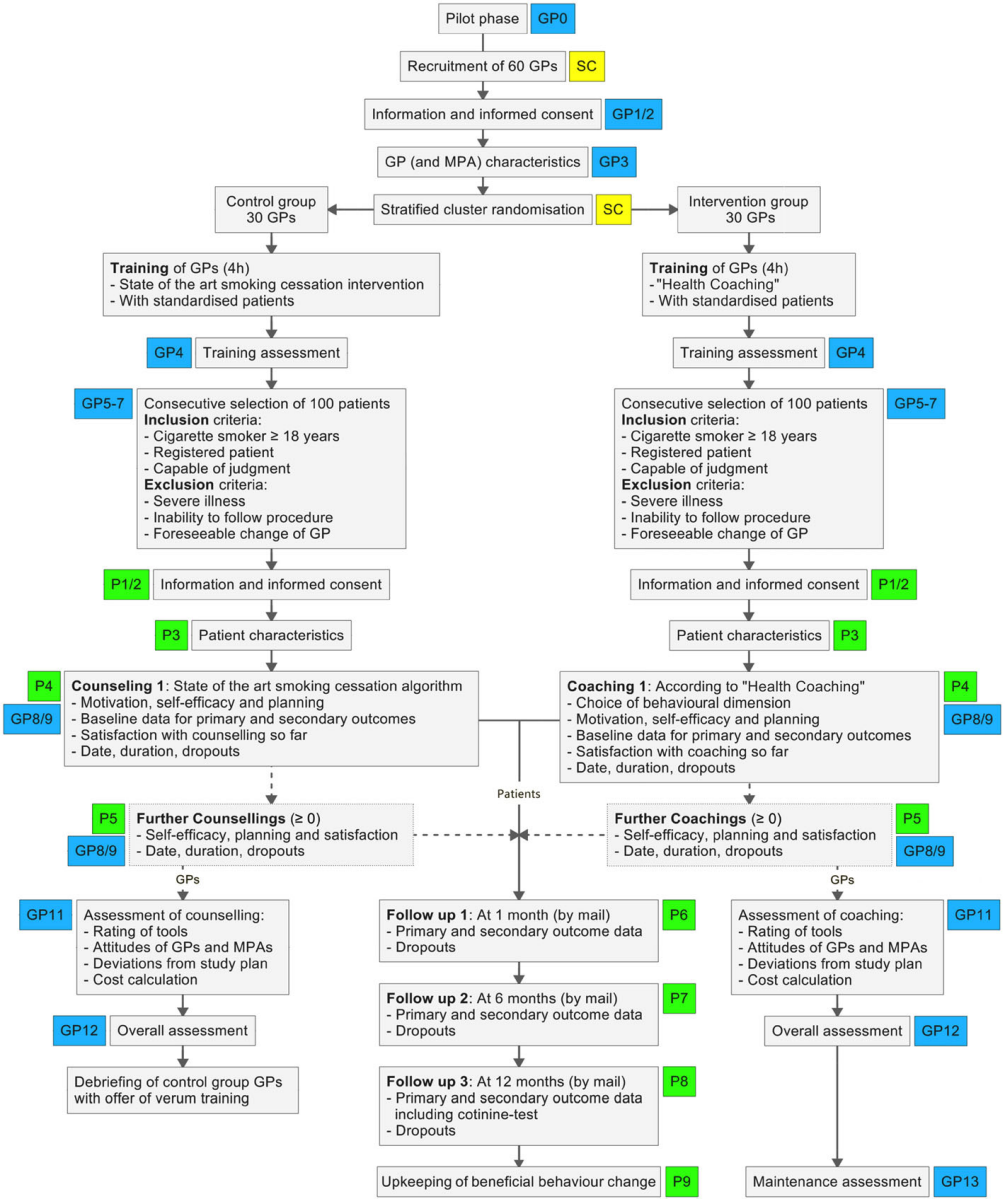
Study flow chart. Small coloured rectangles represent log files and case report forms used to collect data by the study centre (SC) and from general practitioners (GP) and patients (P). MPA medical assistant (medizinische Praxisassistentin)

A cluster randomised controlled trial (randomisation on the GP level) will be conducted with 60 German-speaking GPs from the northern part of Switzerland (see Fig. 1 for the study flow chart). The design and methodology of the study are partly based on experiences gained from a previous trial carried out by the last author in eastern Switzerland [8]. The SPIRIT checklist for this protocol can be found in Additional file 1.

### Eligibility and recruitment of general practitioners

All interested GPs working in outpatient primary care in the German-speaking part of Switzerland are eligible to take part in the planned study, irrespective of their contract status, their experience in primary care, their age, sex or other specifics or affiliations. Included GPs must not have had any previous training in the Health Coaching programme.

In a first step, all members of an interested network of GPs will be invited through a formal letter from the Institute of Primary Care of the University of Zurich. In case of insufficient response, email remainders will be sent out before other doctors’ networks will be included in the recruitment process, and addresses of potential participants from existing in-house and freely accessible federal databases of primary care providers will be considered.

### Cluster randomisation

Participating GPs will be listed chronologically, and GP characteristics collected prior to their allocation to either intervention or control arm. Until their required number is reached (according to the sample size calculation), the GPs will be allocated to the two equally sized study arms in blocks of 14–20 GPs each, by means of a tailored covariate constrained randomisation procedure [9] with an additional constraining criterion regarding potential contamination. In some more detail, a set of sufficiently balanced allocations with regard to the GPs' sex, contract status and practice type will be constructed and then purged from contamination-prone allocations (i.e. from those which put GPs belonging to the same group practice into different study arms), before randomly drawing the final allocation to be used in the study. All GPs from the same group practice will be allocated within the same block.

The block size was chosen to meet recommendations by Carter and Hood [10] and also with a view to training group sizes that can well be handled within the study centre’s resources. To generate the set of balanced allocations, we will use the web-based Shiny Balancer [11], and the additional reduction step will be carried out manually using Excel’s sorting and filtering capabilities. The final random draw will be carried out by a study nurse not involved in the later data analysis.

### Patient recruitment and allocation

All GPs will be asked to approach, consecutively and regardless of the reason for the consultation, current cigarette smokers at least 18 years old. By insisting on consecutive targeting of *all* smokers, we aim to eliminate selection bias very likely to arise if GPs were allowed to select patients whom they deem suitable. In order to include a total of 200 patients, each of the 60 GPs is expected to recruit three to four patients on average.

Patient inclusion criteria:

- Current cigarette smoker.
- Male or female over 18 years of age.
- Registered as patient in the recruiting GP’s patient base.
- Capable of judgement with regard to participation in the study.
- Signed informed consent after being informed.

Patient exclusion criteria:

- Severe general or psychiatric illness (e.g. malignancy, major depressive episode, dementia, etc.).
- Inability of the participant to follow the procedures of the study due to other reasons (e.g. language problems).
- Foreseeable change of general practitioner within 1 year (e.g. due to planned relocation).

Any participating patient will be assigned to the trial arm of his GP without involving further randomisation, and subsequently a set of baseline characteristics (biological and social traits and particulars of their smoking habits) will be collected from every patient included. Since not all GPs might recruit the exact same number of patients and due to the deterministic allocation of patients, we expect to record—and document—some inevitable imbalance between the trial arms with regard to patient numbers and (patient) baseline characteristics. On the other hand, randomness in the GPs' appointment schedules together with consecutive patient inclusion and coupled with the patients’ blinding with regard to the intervention (see next subsection) will translate into largely randomly composed study clusters. This will prevent extremely imbalanced trial arms.

### Blinding

Considering the nature of the interventions, full blinding of GPs and their patients in the strictest sense is not possible. However, in order to achieve the highest possible degree of double-blinding, both GPs and patients will merely be informed that the intervention is aimed at promoting beneficial health-related behaviour changes among smokers, but neither GPs nor patients will be told which of the two interventions (study or control) they provide or receive. Before consenting to participate, the patients will not be offered detailed information about the intervention they will receive, nor will they know details about the intervention in the respective other trial arm during the ongoing study. For those GPs or patients familiar with clinical studies, a minimum amount of related information will be available from registry databases and from the published protocol, which we consider inevitable.

At the end of the study, GPs will be debriefed and those in the control arm will be offered training in Health Coaching too. Ethical approval has been granted for the blinding procedures.

### Intervention

Both the study and the control intervention consist of a communication training session for the participating GPs, identical in duration and organisation but different in content. Training sessions will take 4 h and involve standardised patients (i.e. actors posing as patients) and case vignettes tailored to health-related behaviour counselling [12]. The 60 GPs will be split into groups of 7–10, and each group will then be trained within a short interval of time.

The 30 control group GPs will receive training in smoking cessation counselling as put forward by Frei von Tabak [5].

The training of the 30 GPs in the study arm will cover the key elements of the Health Coaching programme, namely patient-centred counselling, shared decision-making, motivational interviewing and the use of validated tools from existing health promotion programmes such as PAPRICA—Physical Activity Promotion in Primary Care [13], Brief Interventions in Patients with Risky Alcohol Consumption [14] and aTavola (a short intervention programme for healthy eating) [15]. Like their counterparts in the control group, the GPs in the intervention group will also be trained in smoking cessation counselling according to Frei von Tabak [5].

The intervention on the patient level will consist of either activating and coaching patients in achieving a beneficial change of their self-selected unhealthy behaviour (study intervention group) or of smoking cessation counselling (control group) in up to three (or occasionally, if necessary, more) sessions. The number of sessions will depend on progress and the needs of the patients and will be decided on by both patients and their physicians. Coaching or counselling sessions can be carried out by medical assistants or nurses provided that they take part in a training course as already described.

### Outcome measures

#### Primary outcome

The primary outcome is the difference between intervention and control group in the proportion of patients with *any relevant health-promoting change* (as defined in Table 1) in either smoking behaviour, body weight, physical activity, alcohol consumption, stress level, eating habits or another self-chosen health-related behavioural dimension. The primary outcome will be collected (including confirmatory cotinine testing) 12 months after baseline, and also as secondary outcomes at 1 and 6 months.

**Table 1:** Definitions of health-relevant behavioural changes

Behaviour dimension: relevance criterion	Measuring method	References
Smoking: abstinence or reduction of daily number of cigarettes by ≥ 50% from a baseline of ≥ 15 cigarettes	Self-declaration, confirmatory saliva cotinine test at 12 months for quitters	[﻿18﻿, 27]
Body weight: reduction by ≥ 5% if baseline-BMI ≥ 25 kg/m^2^	Standardised home measurements	[28–30, 45, 46]
Physical activity: increase of MVPA by ≥ 90 min per week or increase of LIPA by ≥ 200 min per week, compared to baseline	Recollection-based self-declaration in questionnaire	[31–33]
Alcohol: reduction in number of standard drinks (10 g) per week by ≥ 7 drinks from a baseline of ≥ 14 drinks/week	Recollection-based self-declaration in questionnaire	[35, 36, 47]
Stress: reduction in score of the Perceived Stress Scale (PSS-10, German version) by ≥ 5, compared to baseline	Recollection-based self-declaration in validated questionnaire	[38–41]
Diet: increase by ≥ 10 in score of adapted MedDietScore questionnaire, compared to baseline	Recollection-based self-declaration in validated questionnaire	[44, 48]
Participant’s choice: increase by ≥ 2 levels on a 5 level Likert-type scale (−/0/+/++/+++)	Self-declaration in questionnaire	–

*BMI* body mass index, *LIPA* light-intensity physical activity, *MVPA* moderate- to vigorous-intensity physical activity

The chosen criteria each reflect the lowest level (cut-off point) of a behavioural change with a proven health benefit. For rationales behind the choice of these levels and a justification for choosing “any change” as the binary primary outcome, see the Discussion section.

#### Secondary outcomes

Additional secondary outcomes are presented in Table 2. All secondary outcomes will be compiled at 1, 6 and 12 months after baseline from the same data already collected for the primary outcome, with the exception of self-efficacy as well as action and coping planning which are collected during the ongoing counselling on Likert-type scales with five levels.

**Table 2:** Secondary outcomes

Secondary outcome	
Smoking cessation rates in intervention and control groups among patients with *high intrinsic motivation* to stop smoking	
Smoking cessation rates in intervention and control groups among *all* participants	
Reduction in number of cigarettes per day, compared to baseline	
Weight loss in units of 1 kg, compared to baseline	
Increase in physical activity time per week in units of 5 min, compared to baseline	
Reduction in number of standard drinks per week, compared to baseline, and number of alcohol-free days per week	
Reduction in score of the Perceived Stress Scale (PSS-10, German version), compared to baseline	
Increase in score of translated MedDietScore questionnaire, compared to baseline	
Patients’ degrees of motivation and, if applicable, confidence to achieve and maintain a change in behaviour (self-efficacy)	
If applicable: availability of a plan on when and how to take action (action planning) and existence of a relapse plan (coping planning)	

Additionally, the covariates presented in Table 3 are collected, if applicable.

**Table 3:** Covariates

Covariate	Source
Biopsychosocial data of general practitioners (age, gender, experience, type of doctor’s practice, data of medical assistants/nurses)	GP
Biopsychosocial data of patients (age, gender, marital status, educational level, smoking status of partner)	Patient
Characterisation of smoking behaviour (age at onset of smoking, pack-years, number of cigarettes per day, time to first cigarette after wake up, number of previous attempts to stop smoking)	Patient
Participants’ choice of behavioural dimension	GP
Number and duration of coaching/counselling sessions (per patient and in total)	GP
Person conducting the coaching/counselling (doctor or specifically trained medical assistant)	GP
Type of smoking cessation intervention used	GP
Dispense of decision aids	GP
Involvement of partner or peers into the coaching/counselling process	GP
Perceived partner or peer support	Patient
Perceived willingness of partner or peers to achieve the same change in behaviour	Patient

*GP* general practitioner

#### Process outcomes

In parallel to the ongoing study, additional data will be collected regarding the implementation, effectiveness and cost–benefit ratio of the intervention with the aim of forming a solid basis for decision about further promotion and dissemination of the Health Coaching programme. The results of the evaluation are expected to lead to more profound insights into the methodology of the intervention, in how to optimally train the multiplicators and into factors influencing the acceptance of the intervention by the target population. Furthermore, the process evaluation comprises an analysis of the costs generated by the intervention.

We chose the RE-AIM framework [16] by Glasgow et al. [17] as a comprehensive concept within which to address these objectives.

Process outcomes (Table 4) will be evaluated based on case report and log files used within the actual trial and also using additional methodology specific to the respective questions: semi-structured interviews, questionnaires, telephone interviews and focus group sessions.

**Table 4:** Process outcomes

Process evaluation outcome	Source
Reach: proportion and representativeness of individuals receiving the intervention (individual level)	
Rate of smokers among the GP's patients	GP
Rate of smokers invited to participate	GP
Rate of refusals, with reasons	GP
Patient dropout rate, with reasons	GP
Patients’ characteristics and representativeness	See covariates
Efficacy: success rates of the intervention (including satisfaction outcomes) under study conditions (individual level)	
Behaviour change rates	See outcomes
Changes in self-efficacy and planning	See outcomes
Patients’ current overall satisfaction with coaching programme	Patient
Adoption: proportion and representativeness of organisations willing to adopt the intervention, with consideration of enablers and barriers to adoption (organisational level)	
Rate of GPs invited to participate	SC
Rate of refusals, with reasons	SC
GP dropout rate, with reasons	SC
GPs' characteristics and representativeness	See covariates
Involvement of medical assistants/nurses	See covariates
Assistants'/nurses' characteristics	See covariates
Assessment of GPs' precognitions, understanding of coaching/counselling concept and increase in knowledge and skills, evaluation/rating of coaching/counselling concept and structure of training, recommendation to colleagues	GP
Time required for coaching/counselling	See covariates
Rating of coaching/counselling tools in matters of usefulness and manageability, enabling factors and barriers in coaching/counselling as perceived from practical experience	GP
Overall assessment: benefit for daily practical work, most crucial aspects (success factors and pitfalls), suggestions for improvement, overall satisfaction	GP
Implementation: extent to which the intervention is implemented under real-world conditions—patient adherence (individual level) and adherence of staff to study protocol (organisational level)	
Number of coaching/counselling sessions per patient	See covariates
GPs'/assistants'/nurses' attitudes, competences and (mental) barriers towards/in/to delivering the interventions	GP
Changes in contents or duration of coaching/counselling elements during the ongoing trial	GP
Completeness/integrity of data reported by GPs	SC
Costs arising from expenditure of coaching time	SC
Maintenance: sustainability of intervention over time—relapse rates (individual level) and integration of intervention into institutional routine (organisational level)	
Upholding of beneficial behaviour at 12 months after end of follow up	Patient
Number of GPs trained in HC until 12 months after end of follow up	HC registry
Number/frequency and quality/intensity of coaching over a period of 12 months beyond follow up	GP
Response among experts and media coverage	Experts, media
Maintenance costs	GP

*GP* general practitioner, *HC* Health Coaching programme, *SC* study centre

#### Safety outcomes

Adverse events are defined as any untoward medical occurrences in patients after the intervention and do not necessarily have a causal relationship with study activities. In particular, the following will be considered as adverse events:

- Complication of an existing disease.
- Onset of new acute illness.
- Hospitalisation or death.

Adverse events will have to be recorded by the GPs in the patients’ case report files and reported to the study centre. The study investigators will decide together with the respective GP whether there is a plausible connection between intervention and adverse event.

### Follow up

At 1, 6 and 12 months post baseline the patients will report in mail form about their health behaviour status (regarding smoking, weight, activity, alcohol, diet, stress and—if applicable—self-chosen behaviour dimension). Patients claiming to have achieved tobacco abstinence at 12 months after baseline will also be asked to provide a saliva sample for confirmatory cotinine testing.

In addition to a base compensation, the GPs will be rewarded with financial compensation for every patient providing full follow-up data.

### Sample size calculations

For sample size calculations with respect to the superiority hypothesis, we assume success rates of 40% in the intervention and 15% in the control group. The 40% assumption is based on results from the preceding study [8] in which one out of two participants declared subjective improvements in their self-chosen behavioural dimension after coaching. The success rate of 15% in the control group is the sum of a 10% smoking cessation rate achievable by state-of-the-art smoking cessation counselling according to Comuz et al. [18] plus an estimated 5% of “spontaneous” effects of smoking cessation counselling on one of the other behavioural dimensions. Using *α* = 5% (two-sided) and *β* = 10% we calculated a sample size of 62 participants per equally sized study arm [19] or 2 · 62 / (1 – 0.25) = 166 participating smokers in total after factoring in a dropout rate of 25%. To reflect the correlation structure of the clustered design, an intra-cluster correlation coefficient (ICC) of 4% was assumed, which is slightly higher than corresponding values suggested by Parker et al. [20], in order to account for rare cases of GPs from the same group practice. This assumption is also backed by (unpublished) calculations from the previous study [8] with similar cluster structure and outcome. Correction for the cluster effect increases the sample size to 166 · [1 + (4 – 1) · 0.04] = 186 or roughly 200 patients, assuming that a general practitioner is able to recruit four patients. Finally, allowing for a dropout rate of 15% among general practitioners, 186 / 4 / (1 – 0.15) = 56 (rounded to the next higher even number) or roughly 60 general practitioners must be recruited to detect the expected difference in the primary outcome between the two study arms.

Sample size calculations with respect to the non-inferiority hypothesis require additional assumptions. In the earlier study [8], 12% of smoking or non-smoking GP patients chose smoking cessation as their primary goal. For the present study with smoking participants only, we cautiously assume that in both study arms 25% (i.e. roughly double this number) are highly motivated to give up smoking. Based on Lindson-Hawley et al. [21], we further assume a 40% success rate among the highly motivated. Using a tentative and rather large non-inferiority margin of 10% as in [21] and *β* = 20%, this results in an uncorrected sample size of 297 highly motivated participants per study arm [22] or 2 · 297 / (1 – 0.25) · [1 + (4 – 1) · 0.04] · 4 = 3550 participants in total after correction for clustering and dropouts. Sample size calculations with regard to smoking cessation rates among *all* participants lead to even larger samples, not feasible with available resources. Accordingly, we chose “any relevant behaviour change” as the primary outcome and will consider smoking cessation efficacy among the secondary outcomes.

### Data collection procedures

Patients will receive detailed written information on the aim of the study. After obtaining their written informed consent, data will then be collected by means of paper case report forms (CRFs). For every patient, a dossier with the different CRFs will be created. The forms will be encoded and the codes stored at each GP’s practice. Decoding will be possible if case-tracking is needed (in case of adverse events). Data transfer from paper to electronic form will be carried out and double-checked independently by different research associates.

### Statistical analysis

Baseline characteristics (qualitative and quantitative variables) of GPs and patients will be calculated after recruitment is completed for each arm of the study, with corresponding 95% confidence intervals where applicable.

Crude success rates (i.e. rates of any relevant health-promoting behavioural change and smoking cessation rates) will be compared between intervention and control groups using χ^2^-tests after completion of follow up. As the main analysis, adjustment for cluster effects will then be performed by (multivariate) hierarchical logistic regression with individuals as the unit of analysis, grouping by GP as the random effect and covariates (including the balancing variables) as independent fixed effects.

Changes in outcome measures that do not meet the criteria for clinical relevance defined in Table 1 will be described and presented graphically (density plots, histograms), compared across study arms using χ^2^-tests or *t*-tests and adjusted by random-effects regression models analogous to those used for success rate comparisons, but with the respective behavioural change measures as dependent variables.

All analyses will be conducted following an intention-to-treat (ITT) approach (i.e. outcome data will be obtained from all participants and analysed “as randomised”). Missing values will be replaced by standard multiple imputation (MI) as recommended by Ma et al. [23] for clustered designs with variance inflation factors < 3. Non-responder imputation (NRI; i.e. conservatively treating all missing values as failures) will be used within the scope of sensitivity analyses.

Qualitative information from the process evaluation will be analysed using common coding techniques for qualitative data. A coding tree will be established deductively based on pre-determined domains of interest and refined by inductively adding codes where appropriate. Results will be reported in percentages or categories. Proportions or mean values of different subgroups will be compared using χ^2^-tests or *t*-tests.

### Timeframe

**Fig. 2:**
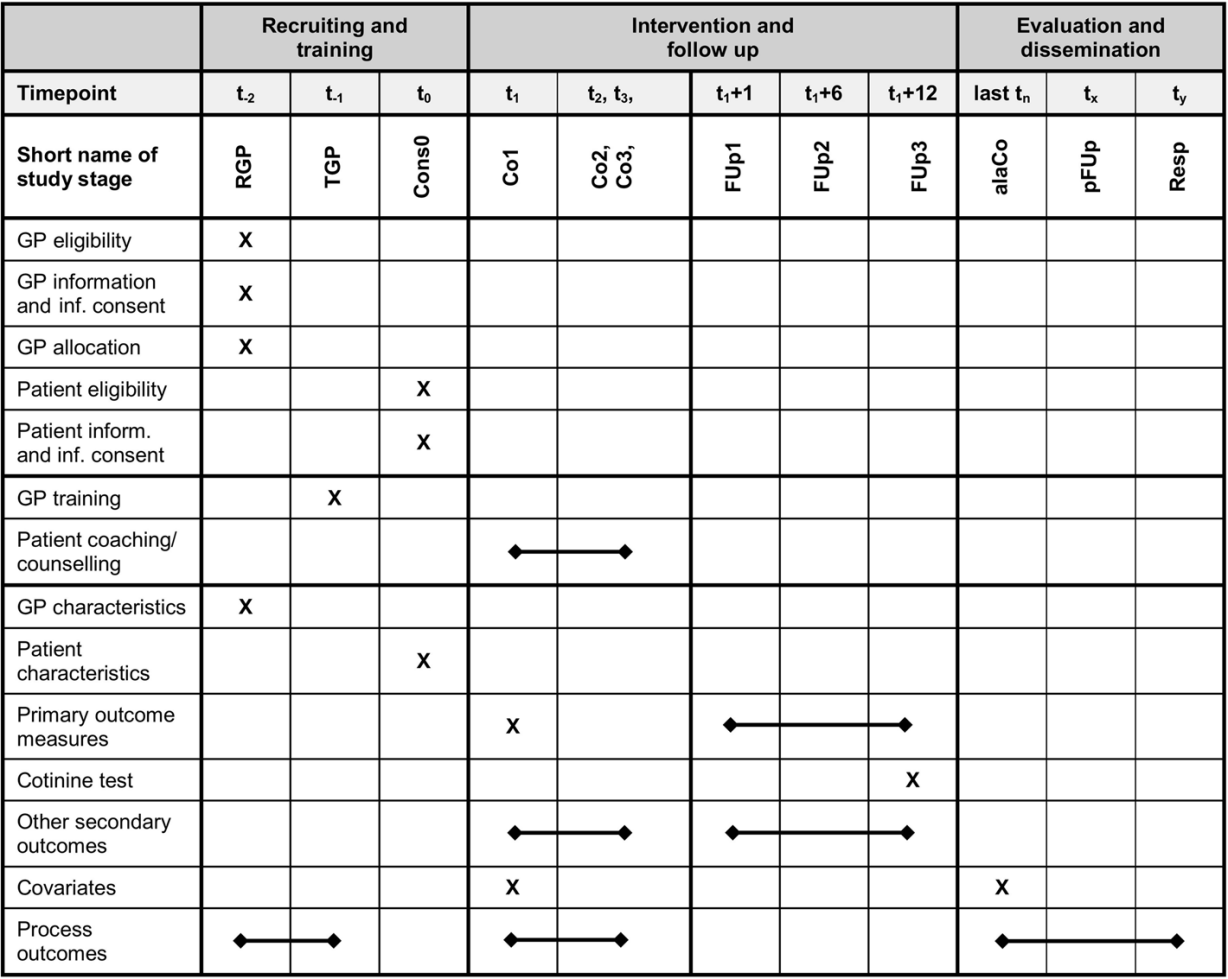
SPIRIT diagram for trial stages of enrolment, intervention and outcome assessment. GP general practitioner, RGP recruiting of GPs, TGP training of GPs, Cons0 consultation 0, Co1–3 coaching/counselling 1–3, FUp1–3 follow up 1–3, alaCo after coaching/counselling of last patient, pFUp post follow up, Resp expert response

Fig. 2 shows for the SPIRIT diagram. The first GP was recruited in mid-2018.  Patients’ eligibility screening and patient inclusion started in the fourth quarter of 2018 with the desired goal of including an average of about one patient per month per GP. Follow up will last for 12 months for each patient.

### Patient informed consent

Previous to study participation, the patients will receive written or verbal information about the content and extent of the planned trial from their individual GPs. In the case of acceptance, they will sign the informed consent form.

### Data security/disclosure of original documents

The patient names and all other confidential information fall under medical confidentiality rules and will be treated according to appropriate Federal Data Security Laws. For contact maintenance and case tracking (e.g. in the case of adverse events), the patients’ identities will be known to a study nurse not involved in the analysis of the patient data. The patient names will not be accessible to the scientific study staff.

## Discussion

### Justification of the binary primary outcome

#### Any change

To our knowledge, the suggested composite outcome has not been used or validated before. It is largely unknown how—in terms of health benefits—a change in some behaviour dimension A compares with a change in another dimension B, and we know of no standardisation procedure to make sizes of changes comparable over different dimensions. To make matters worse, different behaviour changes are measured on different scales, and health benefits have been associated with absolute changes in some measures but with relative changes in others. We believe that, given these imponderabilities, the binary outcome “any relevant change in any component dimension” keeps the utmost possible intrinsic validity, since, firstly, the validity of the individual relevance criteria has been demonstrated and, secondly, no further assumptions on interaction or comparability of different behaviour changes are needed. Moreover, while using a dichotomous primary outcome measure inevitably incurs some loss of information, the study findings will be all the more meaningful for clinical care, provided that a statistical difference will be found [24]. The loss of information will be compensated for by analysing secondary outcomes.

#### Time aspects

Evaluating primary and secondary outcomes at 1, 6 and 12 months after baseline will allow capturing both easy-to-achieve early and hard-to-achieve late behavioural changes. The first two points of time were chosen in accordance with Lindson-Hawley et al. [21] and the last two correspond to the Russel Standard [25] for smoking cessation trials. Data collection at three different times will also provide information on the sustainability of beneficial behaviour changes at the level of the study collective, although not at the individual level. Duration of upkeep of beneficial behaviour patterns was not included among the primary or secondary outcomes because we do not know whether, for example, one sustained change is preferable over two different changes of short duration. However, we will assess the persistence of individual successes beyond follow up as part of the process evaluation.

### Rationales for the choice of relevance criteria

#### Smoking

Within the present study, we consider halving the daily number of cigarettes as sufficiently associated with health benefits. We realise that the evidence supporting this criterion is not overwhelming, and there might even be no benefit in terms of mortality at all [26]. On the other hand, the rate of smoking-related cancers or lung cancers was considerably lower even in Tverdal and Bjartveit’s study [26], although not significantly so as in Godtfredsen et al. [27]. The two studies are somewhat contradicting in terms of size and significance of the effect, which suggests that a reduction by 50% might represent the limit of detection of health benefits from smoking reduction.

#### Body weight

A pro-rata weight loss of 5% is widely accepted as “clinically relevant” or “clinically significant”, see for example Stevens et al. [28], and numerous studies have been carried out using this criterion [29, 30]. Swift et al. [29], for example, demonstrated a significant beneficial effect on insulin levels whereas other cardiovascular risk factors showed short of significant changes in the desirable directions after clinically significant weight loss due to a weight reduction intervention.

#### Physical activity

Current guidelines recommend a minimum of 150 min of physical activity per week of at least moderate intensity (corresponding to ≥ 7.5 MET∙h). Beneficial health effects have been satisfactorily demonstrated for roughly 60% of this amount, for example by Wen et al. [31]. See also the recommendations of the US Physical Activity Guidelines Advisory Committee [32]. According to both sources, additional health effects have been shown for further activity increases by the same amount. Equivalent amounts or equivalent *additional* amounts of physical activity can be achieved with similarly beneficial results at lower intensities but with longer duration [33].

The obvious and tempting idea of using tracking devices had to be discarded on the grounds of practicability (distribution and maintenance of the devices), methodological considerations (recording of baseline activity before the first coaching or counselling session) and due to the sheer costs of providing appropriate sensors to all 200 participants. Out of consideration for the participants (who need to document their health behaviour in all seven dimensions) we also decided against relying on diaries to capture the amount of physical activity. This decision is backed by Timperio et al. [34], who point out that the use of physical activity logbooks does not increase estimates of validity of 7-day recall physical activity questionnaires.

#### Alcohol consumption

The cardioprotective role of alcohol consumption has most probably been overestimated in the past. Recent research, for example, by Rehm et al. [35] suggests a dose–risk relationship with a less pronounced J-shape compared to older results. According to Rehm et al., health risks exceed benefits for any amount of alcohol consumed beyond 10 g per day, irrespective of sex and age. The 2000 WHO guidelines [36] show increasing all-cause mortality estimates for intake classes starting from 10 g/day with class widths of 10 g/day. Our choice of criterion reflects both the cut-off point as well as this magnitude of change, but is based on a weekly assessment as used in most current guidelines on alcohol consumption.

We decided against extending the criterion to cover drink-free days because their benefit is still highly debatable and probably dependent on the total amount consumed [37]. In accordance with measuring physical activity and because the effort was again not deemed reasonable for the patients, we do not intend to use diaries or logbooks that would have to be filled in over several days.

#### Stress level

The Perceived Stress Scale (PSS-10) [38] was chosen for its widespread recognition and frequent use in psychometric research, for the availability of a free and validated German translation, and for its briefness and practicability. Furthermore, the PSS-10 is one of only few scales for which we were able to find satisfactory data describing quantitatively the score changes after stress relief interventions or the association between changes of the score and health outcomes.

Two relatively small studies by Wiegand et al. [39] and Kirby et al. [40], both carried out in non-clinical community settings and with baseline PSS-10 scores of approximately 24 (scale ranges from 0 to 40), found mean decreases in the total PSS-10 scores by about 6.3–9 after stress management interventions of 14 weeks or 10 days, respectively, and about 4.1–5 in the control groups. Based on these data we consider a score reduction by at least 5 as a relevant change. This choice gets some empirical support from results of validation studies that established standard deviations in the order of our criterion [41] and showed that our criterion roughly corresponds to the mean difference between psychiatric outpatients and the general population [42].

#### Eating habits

Concerning food intake there is a vast number of short dietary assessment instruments and screeners available [43] but none of them has prevailed as a generally recognised standard to capture overall diet quality in the primary care setting. The ideal tool was expected to cover the patient’s diet as a whole and without including food items unusual in a typical “local” diet. Furthermore, it was required that laypeople would be able to complete it within reasonable time and without expert knowledge, for example, about different nutritional components of various foods. Lastly, and if at all possible, it had to be validated with respect to some quantifiable morbidity or mortality outcome. The MedDietScore [44] meets these requirements best.

Again, we do not intend to use food diaries for similar reasons as mentioned earlier with respect to physical activity and alcohol consumption. Moreover, we will not try and assess eating habits beyond the mere composition of the diet because we do not know of any validated tools to cover both composition and additional aspects.

### Strengths and limitations

The pragmatic study design allows for recognising beneficial changes in any health-related behaviour. However, there are some important limitations. The necessity to collect data regarding seven different behaviour dimensions requires a significant effort on the part of the participants. Overstraining their cooperation would most likely result in losses to follow up and poor data quality. In the context of the present study, it is therefore not possible to capture all behaviour changes with the highest desirable objectivity. Even though validated measuring instruments will be used as far as possible, recall and desirability bias as well as interpretation bias cannot fully be ruled out. Moreover, some of the chosen cut-off levels for clinical relevance are somewhat debatable. Lastly, we will not be able to completely avoid the selection of suitable cases either on the level of GPs or on the level of patients, even though the consecutive recruitment of patients will prevent the selection of “good risks” by their GPs up to a certain extent.

### Desired impact

The knowledge gained from this study is likely to influence the way GPs deal with harmful behaviour patterns of their patients who smoke. It has the potential to increase care provision for morbidity and mortality risk behaviour beyond mere smoking cessation support. If shown to be effective and feasible, a coaching strategy as set out in the Health Coaching programme and adopted in this study, focusing on shared decision-making and the promotion of the patients’ intrinsic motivation to tackle their individual health problems, could ultimately reduce the high prevalence of risk behaviours in the smoking population.

## Trial status

Patient recruitment had not yet started at the time of the first submission in February 2018 and is planned to take part from late 2018 until mid-2019.

## Additional file

Additional file 1: SPIRIT checklist: recommended items to address in a clinical trial protocol. (PDF 57 KB)

## Abbreviations

CRF: Case report form
GP: General practitioner
ICC: Intra-cluster correlation coefficient
